# Sex influences the effects of social status on socioemotional behavior and serotonin neurochemistry in rhesus monkeys

**DOI:** 10.1186/s13293-023-00562-3

**Published:** 2023-10-28

**Authors:** Alison Wakeford, Jonathon A. Nye, Zachary A. Grieb, Dené A. Voisin, Jiyoung Mun, Kim L. Huhman, Elliott Albers, Vasiliki Michopoulos

**Affiliations:** 1 Emory National Primate Research Center, Atlanta, GA 30322 USA; 2 Department of Radiology and Radiological Science, Medical University of South Carolina, Charleston, SC USA; 3 Neuroscience Institute, Georgia State University, Atlanta, GA USA; 4 Center for Behavioral Neuroscience, Atlanta, GA USA; 5 Department of Psychiatry and Behavioral Sciences, Emory University School of Medicine, Atlanta, GA USA

## Abstract

**Background:**

Despite observed sex differences in the prevalence of stress-related psychiatric conditions, most preclinical and translational studies have only included male subjects. Therefore, it has not been possible to effectively assess how sex interacts with other psychosocial risk factors to impact the etiology and maintenance of stress-related psychopathology. One psychosocial factor that interacts with sex to impact risk for stress-related behavioral and physiological deficits is social dominance. The current study was designed to assess sex differences in the effects of social status on socioemotional behavior and serotonin neurochemistry in socially housed rhesus monkeys. We hypothesized that sex and social status interact to influence socioemotional behaviors as well as serotonin 1A receptor binding potential (5HT1AR-BP) in regions of interest (ROIs) implicated in socioemotional behavior.

**Methods:**

Behavioral observations were conducted in gonadally intact adult female (*n* = 14) and male (*n* = 13) rhesus monkeys. 5HT1AR-BP was assessed via positron emission tomography using 4-(2ʹ-Methoxyphenyl)-1-[2ʹ-(*N*-2ʺ-pyridinyl)-*p*[^18^F]fluorobenzamido]ethylpiperazine ([^18^F]MPPF).

**Results:**

Aggression emitted was greater in dominant compared to subordinate animals, regardless of sex. Submission emitted was significantly greater in subordinate versus dominant animals and greater in females than males. Affiliative behaviors emitted were not impacted by sex, status, or their interaction. Anxiety-like behavior emitted was significantly greater in females than in males regardless of social status. Hypothalamic 5HT1AR-BP was significantly greater in females than in males, regardless of social status. 5HT1AR-BP in the dentate gyrus of the hippocampus was significantly impacted by a sex by status interaction whereby 5HT1AR-BP in the dentate gyrus was greater in dominant compared to subordinate females but was not different between dominant and subordinate males. There were no effects of sex, status, or their interaction on 5HT1AR-BP in the DRN and in the regions of the PFC studied.

**Conclusions:**

These data have important implications for the treatment of stress-related behavioral health outcomes, as they suggest that sex and social status are important factors to consider in the context of serotonergic drug efficacy.

## Plain language summary

Females are more likely to suffer from stress-related conditions that impact socioemotional behavior compared to males. One thing that influences how sex impacts stress-related health problems is social dominance. We examined whether there are sex differences in the effects of social dominance on socioemotional behavior in socially housed rhesus monkeys. Because the neurotransmitter serotonin is important for socioemotional behavior, we also looked at the levels of the 5HT1AR receptor using neuroimaging. Aggression was greater in dominant compared to subordinate animals, and submission was significantly greater in subordinate versus dominant animals and greater in females than males. Anxiety and levels of 5HT1AR in the hypothalamus were significantly greater in females than in males. 5HT1AR in the hippocampus was greater in dominant compared to subordinate females but was not different between dominant and subordinate males. Overall, these data are important for the treatment of stress-related behavioral health outcomes because suggest that sex and social dominance are important factors to consider in the context of how effective drugs targeting the serotonin system are for treating stress-related behavioral health conditions.

## Highlights

- Investigation of sex and social status impacts risk on socioemotional and serotonin 5HT1A receptor binding potential (5HT1AR-BP) in rhesus monkeys.
- Aggression emitted was greater in dominant compared to subordinate animals, and submission emitted was significantly greater in subordinate versus dominant animals and greater in females than males.
- Anxiety-like behavior and hypothalamic 5HT1AR-BP were significantly greater in females than in males.
- 5HT1AR-BP in the dentate gyrus was greater in dominant compared to subordinate females but was not different between dominant and subordinate males.
- These data have important implications for the treatment of stress-related behavioral health outcomes.

## Introduction

Sex differences in the prevalence of many psychiatric disorders that impact socioemotional behaviors are well-documented in people [1]. Females show higher prevalence rates of many stress-related conditions, including anxiety, depression, and posttraumatic stress disorder (PTSD) [2–5], whereas males show greater prevalence rates of neurodevelopmental disorders, such as attention–deficit/hyperactivity disorder (ADHD) and autism spectrum disorder (ASD) [6]. Despite these observed sex differences in behavioral health conditions, both preclinical and translational, neuroscience-focused studies have largely only included male subjects [7], precluding their ability to effectively assess how sex interacts with other psychosocial risk factors to impact the etiology and maintenance of stress-related psychopathology. The lack of inclusion of females may have contributed to the lack of generalizability of findings to date as well as the lack of evidence-based treatments and interventions for stress-related psychopathology.

One psychosocial factor that interacts with sex to impact risk for stress-related behavioral and physiological deficits is social dominance. Social dominance relationships govern social interactions in many species and serve to maintain stability and attenuate social conflict [8]. Across species, these social dominance relationships are formed and maintained by aggressive behaviors that serve to reduce continual and intense social conflict in groups [8–10]. Importantly, high social dominance increases resilience to the effects of social stressors on the body and brain and decreases vulnerability to stress-related psychiatric disorders [11–14]. Low social rank (e.g., social subordination), on the other hand, results in increased psychological and physiological stress responses, including dysregulation of the hypothalamic pituitary adrenal (HPA) axis and increased systemic inflammation, which underlay increased risk for adverse behavioral and physical health outcomes [15, 16]. Although social dominance and subordination confer resilience and risk, respectively, for stress-related adverse health outcomes, the investigation of how these social factors impact neurobiology to exert these effects has received very limited attention in males and almost no attention in females [17]. This includes studies focused explicitly on the central serotonergic pathways that are implicated in the etiology of stress-related psychiatric conditions [18].

Serotonin (5HT) receptors are expressed in areas of the brain that modulate neuroendocrine responses to stressors (i.e., the hypothalamus and hippocampus), as well as behavioral adaptations in response to stressors (prefrontal cortical areas) [19, 20]. Serotonergic neurotransmission is altered in individuals with stress-related psychiatric conditions, including dysregulated 5HT and its metabolites, including 5-hydroxyindoleacetic acid (5HIAA) [21]. In addition, individuals with depression or anxiety-related disorders have lower 5HT1A receptor (5HT1AR) binding potential (5HT1AR-BP) in comparison to healthy controls [19, 20]. Similarly, 5HT1A receptor binding is decreased in patients with panic disorder or social anxiety disorder [22, 23]. Importantly, these same stress-responsive, serotonergic brain regions are also part of the social decision-making network (SDMN), an evolutionarily conserved neural network including hypothalamic, prefrontal and mesolimbic regions that regulate agonistic behaviors [17, 24, 25]. While 5HT typically acts to inhibit male aggression [26–30], 5HT has recently been shown to stimulate aggression in females. For example, data from Syrian hamsters, a rodent species in which females display competitive aggression and form dominant–subordinate relationships at levels greater than or similar to males [31], acute fluoxetine administration and injections of a selective 5HT1AR agonist into the anterior hypothalamus both result in attenuated aggression in males but augmented aggression in females [17]. In addition, this sex-dependent effect of 5HT on agonistic behavior is further impacted by social status, as fluoxetine administration in hamsters decreases aggression in subordinate but not dominant females and decreases aggression in dominant but not subordinate males [32]. There is also evidence for interactions between sex and social status on 5HT1A receptor binding density in hamster brain [25]. Taken together, these findings indicate that sex and social status are important factors to consider in the context of 5HT’s ability to modulate socioemotional behaviors.

One translational, non-human primate model that has been leveraged to understand the impact of social dominance and subordination on socioemotional behavior is socially housed, female rhesus macaques. Rhesus macaques form and maintain strict, hierarchical dominance relationships between members that are maintained by agonistic interactions that govern social interactions, similar to many other mammalian species [8, 33]. Importantly, social dominance in female rhesus monkeys is associated with resistance to the physiological consequences of social stress, while social subordination is associated with HPA dysfunction and heightened systemic inflammation [13, 34]. Studies of the neurobiological consequences of social subordination in female rhesus monkeys show that 5HT1AR-BP in subordinate animals is decreased in comparison to dominant animals in the hippocampus and hypothalamus [35]. Similarly, cynomolgus macaques demonstrating depressive-like phenotypes exhibit decreases in 5HT1AR-BP in the dorsal raphe, hippocampus, and anterior cingulate cortex [36]. Although these data suggest that social status directly impacts 5HT systems critical for regulating socioemotional behavior in female macaques, it remains unclear how social status influences these outcomes in male macaques. Thus, the current study was designed to assess sex differences in the effects of social status on socioemotional behavior and serotonin neurochemistry in socially housed rhesus monkeys. We hypothesized that sex and social status would interact to influence socioemotional behaviors as well as 5HT1AR-BP in brain regions of interest (ROIs) implicated in the regulation of aggressive, affiliative, and anxiety-like behaviors [12, 17, 35, 37, 38] and impacted by stress-related psychopathology in humans [15, 39]. More specifically, we assessed 5HT1AR-BP in the hypothalamus, the hippocampus, the dorsal raphe nucleus (DRN), and subregions of the PFC, including the frontalorbital cortex, straight gyrus, lateralorbital cortex, and inferior frontal cortex.

## Materials and methods

### Subjects

Gonadally intact, unrelated adult female (*n* = 14; average ± SD age: 196 ± 25.9 months) and male (*n* = 13; average ± SD age: 74.9 ± 21.9 months) rhesus monkeys housed in six social groups of three to six monkeys each were subjects in the current study. Social groups were comprised exclusively of same sex membership (only females with females and males with males). Animals were housed in indoor–outdoors runs (3.7 m × 3.7 m × 3.7 m) at the Emory National Primate Research Center (ENPRC) in Lawrenceville, Georgia. Animals were maintained on a commercially available Purina monkey chow diet (5038) ad libitum and had continuous access to water. Seasonal fruits and vegetables were provided daily as a nutritional supplement. The Emory University Institutional Animal Care and Use Committee approved all procedures in accordance with the Animal Welfare Act and the U.S. Department of Health and Human Services “Guide for Care and Use of Laboratory Animals.”

Social groups of females and males had been established for at least three years as described previously [40] and were studied for five weeks in the current study during the fall breeding season (October–January) [41]. The social rank of animals within each group was established by the outcome of dyadic agonistic interactions in which subordinate animals emit an unequivocal submissive behavior towards other animals in their groups [8, 42]. These social ranks based on dyadic agonistic interactions were verified by the calculation of David’s Scores and the steepness for each social group hierarchy was calculated using the *EloRating* package in R (v24) [43, 44]. Animals ranked as 1 and 2 were categorized as dominant, and animals ranked 3–6 were classified as subordinate in accordance with previously established conventions [13, 14, 45]. Based on this definition of social status, we studied six dominant and eight subordinate females, and six dominant and seven subordinate males. While females were older than males (*p* < 0.001) and female hierarchy steepness were less steep than those of the males (*p* < 0.001), group size (*p* = 0.88) and David’s scores (*p* = 0.98) were not different between the sexes. Table 1 describes the group size, age, steepness and average behavioral rates for each of the sex groups of animals studied in the current study.

**Table 1:** Mean ± SEM of age, social group information, and rates of aggressive, submissive, affiliative, and anxiety-like behavior (per 30 min) for each group of females (F) and males (M)

Group	Sex	Age* (months)	Group size	David’s scores	Hierarchy steepness*	Aggression	Submission	Affiliation	Anxiety-like
1	F	192 ± 9.24	6	0.001 ± 2.26	0.47	14.7 ± 7.05	20.2 ± 19.8	34.8 ± 3.23	113 ± 10.4
2	F	193 ± 11.3	4	< 0.001 ± 2.77	0.48	32.0 ± 8.64	84.0 ± 24.2	45.0 ± 7.63	114 ± 12.7
3	F	203 ± 11.3	4	0.001 ± 2.77	0.58	12.0 ± 8.64	44.3 ± 24.2	38.3 ± 7.63	101 ± 12.7
4	M	74.8 ± 10.1	5	− 0.138 ± 2.47	0.69	3.20 ± 7.73	26.6 ± 21.7	14.0 ± 6.83	41.6 ± 11.3
5	M	101 ± 13.1	3	0.002 ± 3.20	0.72	0.00 ± 9.97	3.67 ± 27.9	13.0 ± 8.81	34.7 ± 14.6
6	M	138 ± 10.1	5	0.002 ± 2.47	0.73	6.00 ± 7.73	38.4 ± 21.7	29.0 ± 6.83	26.8 ± 11.3

Asterisks denote significant sex differences in age and hierarchy steepness (*p*’s < .05)

### Behavioral observations

Behavioral observations were collected for each group using a standard monkey ethogram over five weeks to capture rates of aggression, submission, affiliation, and anxiety-like behavior. Social behavior was captured via five, 30-min observations for each group that were conducted weekly during the study in the afternoon using an established monkey ethogram to create index scores based on counts of individual behaviors per 30 min [40]. Aggression was measured by threats, slaps, grabs, and bites, and submissive behavior was characterized by withdrawals, grimaces, and screams [40]. Affiliative behavior was comprised of engagement in proximity and grooming [40]. Anxiety-like behavior consisted of body shakes, yawns, and self-scratching [46]. Data were recorded using a Windows Laptop and the “Hand Obs” program developed by the Center for Behavioral Neuroscience [47]. Inter-observer reliability was greater than 92%.

### PET neuroimaging

At the end of the 5-week behavioral study period, all subjects underwent PET neuroimaging using 4-(2ʹ-Methoxyphenyl)-1-[2ʹ-(*N*-2ʺ-pyridinyl)-*p*[^18^F]fluorobenzamido]ethylpiperazine ([^18^F]MPPF), which was previously validated in monkeys by our group [48]. PET images were acquired on a microPET Focus 220 scanner system (CTI Concorde Microsystems LLC, Knoxville, TN). [^18^F]MPPF was synthesized by nucleophilic substitution reaction with [^18^F]F^−^, which was produced by ^18^O(p,n)^18^F reaction with a Siemens RDS111 in the ENPRC Imaging Core. PET imaging occurred at the same time of day to control for any diurnal effects [49]. Animal anesthesia (isoflurane 1 to 2% to effect) and monitoring followed standard veterinary practices [35]. A transmission scan was obtained with a cobalt-57 source for attenuation correction of the emission data. [^18^F]MPPF was infused over one minute. Emission data were collected continuously over 120 min from the start of [^18^F]MPPF injection and then binned into appropriate time frames.

### MRI neuroimaging

Structural MR images were obtained within three weeks of the PET scan using a 3 T magnet (Siemens Trio) for evaluation of white matter volumes, and co-registration of PET and delineation of 5HT1AR-BP region(s) of interest (ROIs) using procedures and neuroanatomical definitions previously published in rhesus by our group [48, 50]. Rhesus macaque brain atlases [51–54] were used to guide ROI tracing within structural MRI images in coronal and sagittal views [48]. ROIs included the hypothalamus, the hippocampus, the dorsal raphe nucleus (DRN), and subregions of the PFC, including the frontalorbital cortex, straight gyrus, lateralorbital cortex, and inferior frontal cortex. All these regions have been implicated in 5HT’s actions on socioemotional behavior [12, 17, 35, 37, 38] and are linked to the etiology of stress-related psychopathology [15, 39]. Time-activity curves (TACs) were generated for each ROI and the remaining analysis was performed with in-house software developed by the authors in the International Data Language environment (Harris Geospatial Solutions Inc., Broomfield, CO). Estimates of 5HT1AR-BP were calculated employing established kinetic modeling approaches using the cerebellum as the reference tissue input function [55] in accordance with our previously published protocols [48].

### Statistical analyses

Raw data were tested for normality using Shapiro–Wilk tests and homogeneity of variance using Levene’s test, and data log transformed for when these assumptions were not met (rates of aggressive, affiliative, and submissive behavior and 5HT1A-BP in the hypothalamus and inferior frontal cortex were transformed). The effects of sex (males vs. females), social status (dominant vs. subordinate) and their interaction on each behavioral index (raw counts of aggression, submission, affiliation and anxiety-like behavior per 30 min) and each ROI were assessed using independent ANCOVAs. These behavioral analyses also included age and group size as covariates. Structural MRIs showed that the ROI volumes (in voxels) were significantly larger in males than in females (*p*’s < 0.05; Table 2), except for the DRN (*p* > 0.05). Because of these sex differences in ROI structural volumes, analyses assessing impacts of sex, status, and their interaction on 5HT1A-BP included age, group size, and structural volume of ROI as covariates. Post-hoc analyses were conducted with Fisher’s least significant difference when necessary and significance was set at *p* ≤ 0.05 for all tests. SPSS v29 was used for all data analysis.

**Table 2:** Mean ± SEM structural ROI volumes (units in voxels) in male and female monkeys

ROI	Females	Males
Hypothalamus	273 ± 4.92	304 ± 6.67*
Hippocampus	399 ± 5.75	443 ± 9.24*
Dorsal Raphe Nucleus (DRN)	10.1 ± 0.45	10.5 ± 0.48
Straight gyrus	534 ± 10.5	601 ± 10.9*
Frontalorbital	1356 ± 24.6	1540 ± 28.6*
Inferiororbital	1310 ± 28.2	1478 ± 26.9*
Lateralorbital	711 ± 13.8	807 ± 15.9*

Asterisks denote significant greater ROI volumes in males compared to females

## Results

### Effects of sex, status, and their interaction on socioemotional behavior

**Fig. 1:**
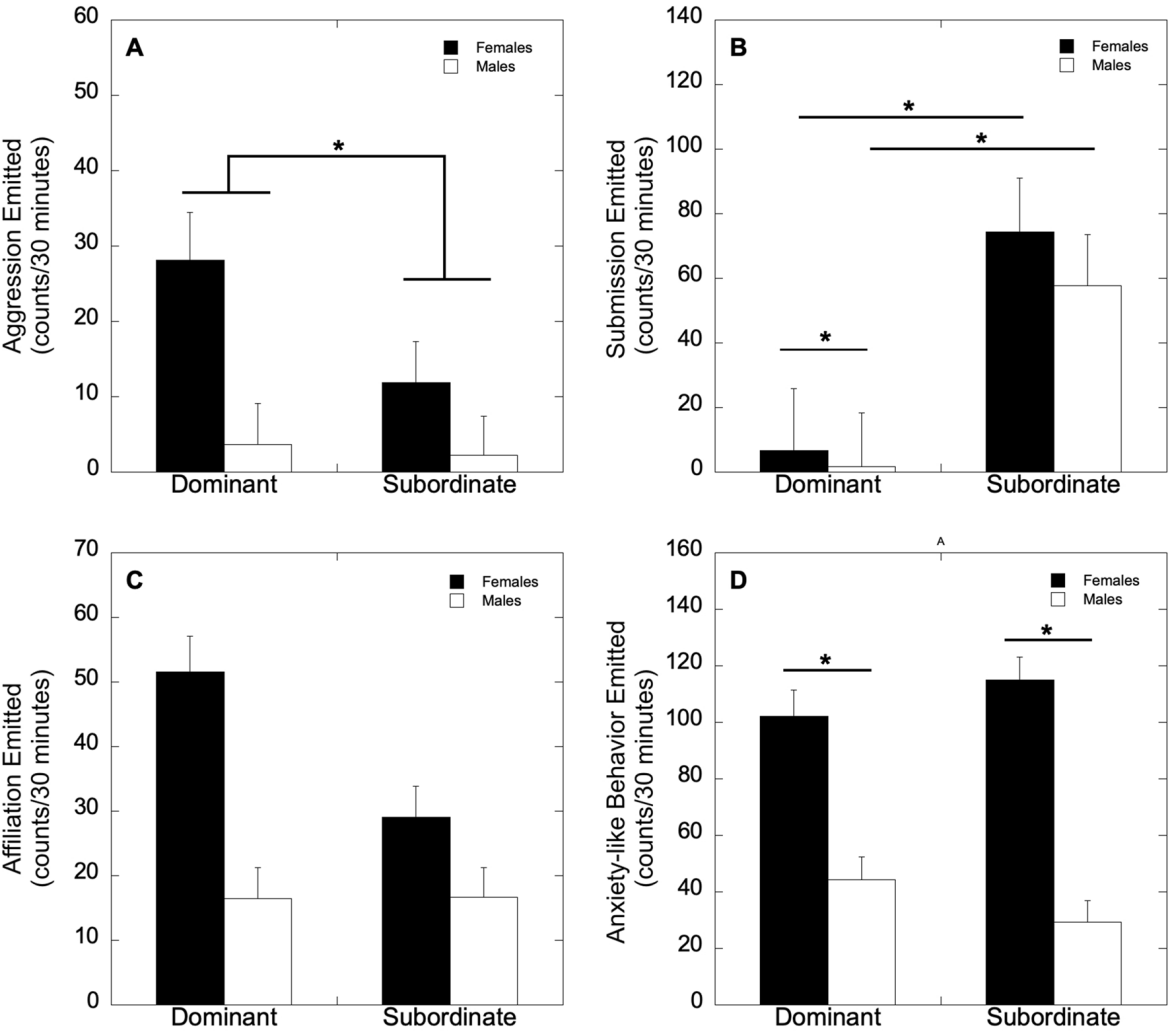
Mean ± SEM frequency of **A** aggression, **B** submission, **C** affiliation, and **D** anxiety-like behavior emitted (counts per 30 min) in dominant and subordinate female and male monkeys. Asterisks denote significant differences between groups of animals (*p*’s < .05)

Aggression emitted was greater in dominant compared to subordinate animals (*F*_1,21_ = 5.17, *p* = 0.034, η^2^ = 0.198; Fig. 1A), regardless of sex (*F*_1,21_ = 1.077, *p* = 0.784). Submission emitted was significantly greater in subordinate versus dominant animals (*F*_1,21_ = 31.8, *p* < 0.001; η^2^ = 0.602; Fig. 1B) and greater in females than males (*F*_1,21_ = 5.06, *p* = 0.035; η^2^ = 0.194; Fig. 1B). Submission emitted was lower in dominant males (*p* < 0.001; Fig. 1B) and females (*p* = 0.003; Fig. 1B) compared to subordinate males and females, respectively. Within dominant animals, submission was greater in females compared to males (*p* = 0.015; Fig. 1B). There were no differences in submission between subordinate females and males (*p* = 0.11; Fig. 1B). Affiliative behaviors emitted were not impacted by sex, status, or their interaction (*p*’s > 0.05; Fig. 1C). Finally, anxiety-like behavior emitted was significantly greater in females than in males (*F*_1,21_ = 5.40, *p* = 0.030, η^2^ = 0.204; Fig. 1D) regardless of social status (*F*_1,21_ = 2.39, *p* = 0.137).

### Effects of sex, status, and their interaction on 5HT1AR-BP

Hypothalamic 5HT1AR-BP was significantly greater in females than in males (*F*_1,20_ = 14.5, *p* = 0.001, η^2^ = 0.420; Table 3) regardless of social status (*F*_1,20_ = 1.24, *p* = 0.278). 5HT1AR-BP in the dentate gyrus of the hippocampus was significantly impacted by a sex by status interaction (*F*_1,20_ = 7.13, *p* = 0.015, η^2^ = 0.263; Table 3). 5HT1AR-BP in the dentate gyrus was greater in dominant compared to subordinate females (*p* = 0.033) but was not different between dominant and subordinate males (*p* = 0.163). 5HT1AR-BP in the dentate gyrus was greater in dominant females compared to dominant males (*p* = 0.044), but not different between subordinate females and males (*p* = 0.81). There were no effects of sex, status, or their interaction on 5HT1AR-BP in the DRN and in other regions of the PFC, including the straight gyrus, frontalorbital, inferiorfrontal, and lateralorbital cortices (*p*’s > 0.05; Table 3).

**Table 3:** Mean ± SEM 5HT1A-BP in dominant and subordinate female and male monkeys

	Dominant		Subordinate	
ROI	Females	Males	Females	Males
Hypothalamus	2.47 ± 0.26*	0.36 ± 0.26	2.69 ± 0.28*	0.18 ± 0.30
Hippocampus	6.40 ± 0.55*^,a^	4.21 ± 0.58	5.25 ± 0.61^b^	4.97 ± 0.66
DRN	3.84 ± 1.20	3.27 ± 1.20	5.13 ± 1.27	3.68 ± 1.43
Straight gyrus	8.05 ± 0.93	4.18 ± 0.85	7.12 ± 0.91	3.99 ± 1.05
Frontalorbital	7.31 ± 0.98	3.38 ± 0.96	6.75 ± 1.03	3.92 ± 1.14
Inferiororbital	7.72 ± 1.14	4.31 ± 1.05	7.77 ± 1.13	5.44 ± 1.27
Lateralorbital	6.42 ± 0.96	3.72 ± 0.89	5.84 ± 0.97	4.01 ± 1.09

5HT1A-BP in the hypothalamus was greater in females compared to males (*p* < .001; *). 5HT1AR-BP in the dentate gyrus was greater in dominant compared to subordinate females (*p* = .033; denoted by letters) but was not different between dominant and subordinate males (*p* = .163). 5HT1AR-BP in the dentate gyrus was greater in dominant females compared to dominant males (*p* = .044; *), but not different between subordinate females and males (*p* = .81)

## Discussion

The current study is one of the first to investigate the impact of sex, social status, and their interaction on socioemotional behavior impacted by stress-related psychopathology in both female and male rhesus monkeys. While the data showed no effects of social status, sex, or their interaction on affiliative behaviors in socially housed rhesus monkeys, aggressive behaviors emitted were significantly greater in dominant versus subordinate animals of both sexes. Submission emitted was significantly greater in subordinate versus dominant animals and greater in females than males. There were no differences in submission between subordinate females and males. In addition, anxiety-like behavior was higher in females than in males regardless of social status. PET neuroimaging studies showed that 5HT1AR-BP in the hypothalamus was significantly greater in females than in males regardless of social status. 5HT1AR-BP in the dentate gyrus was greater in dominant compared to subordinate females but was not different between dominant and subordinate males. Taken together, the current data suggest that sex and social status influence variation in socioemotional behavior and 5HT1AR-BP in ROIs impacted by stress and implicated in the etiology of stress-related psychopathology in a behavior and region-specific manner, respectively.

We first examined whether sex interacted with social status to contribute to differences in socioemotional behavior, including aggression, submission, affiliation, and anxiety-like behavior, in captive rhesus monkeys housed in sex-same social groups. The social status of animals within each group was established by the outcome of dyadic agonistic interactions in which subordinate animals emit an unequivocal submissive behavior towards other animals in their groups [8, 42] and verified with David’s scores [43]. Analyses confirmed that there was a main effect of status on aggressive behaviors, with dominant animals of both sexes displaying more aggression than subordinate animals of both sexes. The lack of sex effects on aggression in the current study could be linked to our inability to account for individual differences in gonadal steroid hormones, including estradiol and testosterone, that fluctuate during the breeding season in both female and male macaques and are associated with increases in aggressive behavior in both sexes [56]. Previous studies in ovariectomized, female rhesus monkeys have shown that estradiol replacement to mid-follicular levels increase aggressive behavior in both dominant and subordinate females [57].

Rates of submission were also impacted by social status such that subordinate animals emitted more submission than dominant macaques. This confirms previous reports that lower ranking animals must emit submissive behaviors to terminate or attenuate the probability of receiving aggression from higher ranking animals in their social groups [8]. We also found that dominant females submitted more than dominant males, an effect driven by the second ranked animals in each group since the highest ranking animals do not submit to anyone in their group. This difference in submissive behaviors between the sexes may be due to the sex difference in the characteristics of the dominance hierarchies [58]. Indeed, the steepness of the female groups was significantly less than that of the male groups, indicating that the male groups had steeper dominance hierarchies that are typical of more despotic and intolerant social structures [59–62]. The greater submission emitted by higher ranking dominant females in the current study may be a consequence of a more even distribution of social connectedness [59–62]. Less steep and more tolerant social hierarchies are also associated with less steep rank gradients in affiliation, perhaps driving the lack of any status or sex differences in rates of affiliative behaviors in the current study. While previous literature indicates that other aspects of social context, such as group size and mixed-sex groups, are also important factors that influence rates of agonistic and affiliative behaviors in monkeys [13, 63–65], fluctuations in reproductive hormones (e.g., estradiol, testosterone, and oxytocin) may also be influencing these behaviors in the current study [57, 66].

There was also a significant sex difference in the overall rates of anxiety-like behavior emitted that was not influenced by social status, as females displayed more anxiety-like behavior than did males. This result parallels reports from women indicating that they suffer from higher rates of anxiety-related psychiatric conditions compared to men [1]. We did not observe an impact of social status on anxiety-like behavior in females or males, contributing to other equivocal reports of social status effects on these behaviors in socially housed macaques [13, 67, 68]. Lack of status differences in anxiety-like behavior could be due to individual differences in gonadal steroid levels that are increased in the breeding season in rhesus macaques [56]. In male cynomolgus macaques, greater body weight (more typical of higher ranking males) has been associated with greater anxiety-like behaviors [69]. Contrary to the well-established anxiolytic effects of estradiol [70], testosterone replacement in chemically castrated male macaques appears to be somewhat anxiogenic, increasing anxiety-like behavior to basal levels [71]. In ovariectomized female rhesus monkeys estradiol replacement to mid-follicular levels decreases anxiety-like behaviors in subordinate females in a manner dependent on the polymorphism found in the promoter region of the *SLC6A4* gene that encodes the 5HT transporter (5HTT) that has been linked to risk for stress-related psychopathology in humans [57, 72].

Because 5HT neurochemistry is implicated in the etiology of stress-related psychopathology and is critical for the regulation of socioemotional behavior assessed in the current study, we also characterized the impacts of sex, social status, and their interaction on 5HT1AR-BP in brains areas implicated in these behavioral outcomes. Alternations in 5HT neurochemistry, including lower levels of prefrontal 5HT1AR-BP and reductions in CSF concentrations of 5HIAA, are associated with anxiety [73, 74] and other stress-related psychopathology in people [19–21]. While our data showed greater hypothalamic in females compared to male macaques, previous studies in humans assessing sex and gender differences in 5HT1AR-BP have been equivocal in nature, as both lower and higher levels of hypothalamic 5HT1AR-BP have been described in women compared to men [75–77]. The equivocal nature of these sex and gender-based comparisons in humans could be due to the lack of assessment or control of gonadal steroid hormones that are known to impact 5HT neurochemistry, including 5HT1AR expression and function [35, 78–80]. Greater E2 is associated with greater 5HT1AR-BP in women [75], and replacement of mid-follicular levels of E2 in ovariectomized female rhesus monkeys increases hippocampal and hypothalamic 5HT1AR-BP [35]. These PET neuroimaging findings in women corroborate studies in ovariectomized female macaques and rats showing that E2 replacement reduces 5HT1AR mRNA and protein expression in the dorsal raphe nucleus, hippocampus, and cingulate cortex [78–80].

In the current study, sex impacted the effects of social status on 5HT1AR-BP only in the dentate gyrus of the hippocampus, suggesting region-specificity in these interactive effects on serotonergic neurochemistry. In the hippocampus, 5HT1AR-BP was greater in dominant compared to subordinate females but was not different between dominant and subordinate males. Social subordination and daily stress exposure in women [81] and depression are associated with reduced levels of hippocampal 5HT1AR-BP in mixed-sex samples [82]. Previous reports from our group show that ovariectomized, subordinate females have lower 5HT1AR-BP in the hippocampus as well as lower concentrations of 5HIAA in cerebral spinal fluid compared to dominant females [83]. While the specific role of hippocampal 5HT actions have been implicated in stress-related behavioral responses, the role of 5HT in the hippocampus on modulating prosocial behaviors, where we found that sex impacted the influence of social status, is less clear. Prior studies of free-ranging rhesus macaques show that relationships between 5HIAA and testosterone and their association with sociosexual behaviors are dependent on reproductive seasonality such that increases in testosterone in male macaques during the breeding season are positively correlated with 5HIAA concentrations, which also increase in the breeding season and are associated with greater grooming behaviors [56].

The current study was limited in that study subjects were housed in same-sex social groups. Previous studies in captive and free-ranging macaques suggest that social group composition and the presence of conspecifics can impact the expression of socioemotional behaviors in both females and males [13, 56, 64]. In addition, while we included age as a covariate in our analyses, age may be confounded with sex in the current study, as females were significantly older than males. Although studies in macaques show that social selectiveness increases with aging in females [84, 85] and age influences aggression received by males [86], we did not see sex effects in rates of affiliation and aggression the current study. Another limitation of the current study was that it was conducted exclusively during the breeding season, when levels of gonadal hormones are high and fluctuating [41]. While this reproductive physiological state better mimics female and male humans during their reproductive years, assessments of estradiol and testosterone in future studies are necessary to determine their impact on behavioral and serotonergic outcomes assessed in the current study. In addition, future studies in female and male macaques are necessary to determine how sex, social status, and their interaction impact the ability of serotonergic pharmacological agents to impact the expression of socioemotional behaviors assessed in the current study.

### Perspectives and significance

Overall, the current data show that sex and social status can impact socioemotional behavior and 5HT1A-BP in ROIs important for the regulation of socioemotional behaviors in rhesus monkeys. These findings extend previous studies from female rhesus macaques and male and female hamsters showing that serotonergic pharmacological agents, such as fluoxetine, can have differential effects on socioemotional behaviors that are dependent on sex, social status, and their interaction [32]. Combined, these data have important implications for the treatment of stress-related behavioral health outcomes, as they suggest that sex and social status are important factors to consider in the context of risk for and treatment of stress-related psychiatric conditions.

## Data Availability

The data that support the findings of this study are not openly available due to reasons of sensitivity and are available from the corresponding author upon reasonable request.
